# Efficacy of neoadjuvant endocrine therapy compared with neoadjuvant chemotherapy in pre-menopausal patients with oestrogen receptor-positive and HER2-negative, lymph node-positive breast cancer

**DOI:** 10.1186/s13058-020-01288-5

**Published:** 2020-05-27

**Authors:** Hee Jeong Kim, Woo Chul Noh, Eun Sook Lee, Yong Sik Jung, Lee Su Kim, Wonshik Han, Seok Jin Nam, Gyung -Yub Gong, Hwa Jung Kim, Sei Hyun Ahn

**Affiliations:** 1 Department of Surgery, University of Ulsan College of Medicine, Asan Medical Center, 88 Olympic ro 43 gil, song pa gu, Seoul, 138-736 South Korea; 2 Department of Surgery, Korea Cancer Center Hospital, Korea Institute of Radiological and Medical Sciences, Seoul, South Korea; 3 Department of Surgery, Center for Breast Cancer, Research and Institute and Hospital, National Cancer Center, Goyang, South Korea; 4 Department of Surgery, School of Medicine, Ajou University, Suwon, South Korea; 5 Division of Breast and Endocrine Surgery, Hallym Sacred Heart Hospital, College of Medicine, Hallyum University, Anyang, South Korea; 6 Department of Surgery and Cancer Research Institute, College of Medicine, Seoul National University, Seoul, South Korea; 7 Department of Surgery, Samsung Medical Center, School of Medicine, Sungkyunkwan University, Seoul, South Korea; 8 Department of Pathology, University of Ulsan College of Medicine, Asan Medical Center, Seoul, South Korea; 9 Department of Preventive Medicine, University of Ulsan College of Medicine, Asan Medical Center, Seoul, South Korea

**Keywords:** Neoadjuvant endocrine therapy, Neoadjuvant chemotherapy, Pre-menopausal women, Breast cancer, Clinical response

## Abstract

**Introduction:**

Neoadjuvant endocrine therapy (NET) has demonstrated efficacy in post-menopausal patients with hormone-responsive breast cancer. This trial was designed to compare the efficacy of neoadjuvant chemotherapy (NCT) with NET in pre-menopausal breast cancer.

**Patients and methods:**

In this prospective, randomised, phase III study, oestrogen receptor (ER)-positive, HER2-negative, and lymph node-positive pre-menopausal breast cancer patients were recruited from 7 hospitals in South Korea. Enrolled patients were randomly assigned (1:1) to receive 24 weeks of either NCT or NET with goserelin and tamoxifen. The primary purpose was to evaluate the non-inferiority of NET compared to NCT using clinical response, assessed by MRI. Besides, pathological complete response rate (pCR), changes in Ki-67 expression, breast conservation surgery (BCS) rate, and quality of life were included as secondary endpoints.

**Results:**

A total of 187 patients were assigned to receive NCT (*n* = 95) or NET (*n* = 92), and 87 patients in each group completed treatments. More NCT patients had complete response or partial response than NET patients using MRI (NCT 83.7% vs. NET 52.9%, 95% CI 17.6–44.0, *p* < 0.001) and callipers (NCT 83.9% vs. NET 71.3%, 95% CI 0.4–24.9, *p* = 0.046). Three NCT patients (3.4%) and one NET patient (1.2%) showed pCR (*p* < 0.005). No difference existed in the conversion rate of BCS (13.8% for NCT vs. 11.5% for NET, *p* = 0.531) and Ki-67 change (*p* = 0.114) between the two groups. Nineteen NCT patients had treatment-related grade 3 or worse events compared with none in the NET group.

**Conclusions:**

Better clinical responses were observed in pre-menopausal patients after 24 weeks of NCT compared to those observed after NET.

**Trial registration:**

Clinicaltrials.gov, NCT01622361. Registration June 19, 2012.

## Background

For hormone-responsive breast cancer, the question of which patients can safely be spared adjuvant chemotherapy has been extensively studied. For cases involving hormone receptor-positive tumours, younger patients (< 35 years old) show a poorer prognosis than older patients in terms of tamoxifen resistance [1]. However, this finding does not indicate that young patients should receive chemotherapy. The greater effects of chemotherapy on younger patients are likely to be partially explained by the endocrine effects of chemotherapy on ovarian function. Irrespective of the chemotherapy agent administered, iatrogenic amenorrhea is associated with an increased survival rate in oestrogen receptive (ER)-positive breast cancer [2, 3], and amenorrhea is a surrogate marker for effective treatment in hormone receptor-positive pre-menopausal breast cancer patients [4]. This finding suggests that irrespective of chemotherapy or endocrine therapy, amenorrhea is important for pre-menopausal patients who have an ER-positive, HER2-negative tumour. The trial assigning individualized options for treatment (TAILORx) and the EORTC 10041/BIG 03-04 (MINDACT) studies can identify patients with a low-risk profile, thereby justifying the omission of chemotherapy based on its potentially low benefit [5–8].

Treatment decisions based on demographic characteristics and tumour burden have the potential to overtreat many individuals and undertreat others. The Oncotype DX assay has been studied for lymph node-positive breast cancer patients, and the SWOG 8818 study reported that the low-risk Oncotype DX group gained no additional benefits from chemotherapy [9]. SOFT and TEXT joint analysis showed excellent survival data that included lymph node-positive, ER-positive breast cancer patients who received endocrine therapy alone. These findings suggested that not all lymph node-positive, ER-positive, and HER2-negative tumours require chemotherapy. Apart from the tumour burden, identifying robust biological predictors of benefit is likely to be informative and useful. It has been shown that endocrine therapy, which can substitute for chemotherapy even though there is a high tumour burden in hormone-responsive breast cancer, is effective. The factors that determine chemotherapy sensitivity remain poorly understood and might not entirely overlap with negative predictors of endocrine sensitivity or prognostic determinants [10, 11].

Neoadjuvant treatment has benefits, which include reducing the tumour burden and evaluating the treatment response of the tumour. The response to neoadjuvant chemotherapy (NCT) is diverse and varies according to the intrinsic subtype. ER-positive, HER2-negative breast cancer shows a reduced pathologically complete response (pCR) rate, but better survival than other subtypes. Moreover, pCR is not related to survival in the luminal A subtype [12]. Therefore, the goal of neoadjuvant treatment for hormone-responsive tumours is to evaluate the treatment effects and reduce the tumour size prior to treatment, although it can rarely achieve pCR. Neoadjuvant endocrine therapy (NET) has been mostly studied for post-menopausal breast cancer patients in comparison with tamoxifen and aromatase inhibitors [13–15]. Semiglazov et al. compared NET and chemotherapy for post-menopausal breast cancer patients and found a similar clinical response between the two therapies [16]. The STAGE study was conducted for pre-menopausal breast cancer patients and compared gonadotropin-releasing hormone agonist (GnRHa) with tamoxifen and GnRHa with an aromatase inhibitor [17]. However, no clinical trial has yet compared NCT with NET for pre-menopausal patients.

Therefore, we hypothesised the non-inferior response of NET compared with NCT and reported a phase III clinical trial to compare the response between NCT and NET in pre-menopausal women with ER-positive, HER2-negative, and lymph node-positive breast cancer.

## Methods/design

### Study design

This is a phase III, open-label, prospective, randomised, multicentre, neoadjuvant study of chemotherapy versus endocrine therapy in pre-menopausal patient with hormone-responsive, HER2-negative, lymph node-positive breast cancer [NEST]. Seven centres belonging to the Korean Breast Cancer Society Group (KBCSG-012) participated in this study. The study protocol has been approved by the Korea Food and Drug Administration (KFDA), as well as the institutional review board of every trial centre, and was conducted in accordance with the Declaration of Helsinki, Good Clinical Practice, and the applicable local regulatory requirements on bioethics. For safety issue, the response rate of both groups was planned to be monitored. An independent data monitoring committee (IDMC) was established to monitor the study progress. The IDMC was responsible for deciding whether to continue the study when 66 patients in each treatment group completed their treatment regimens. This trial has been registered in ClinicalTrials.gov as number NCT01622361.

### Patients

Eligible patients included pre-menopausal women with histologically confirmed ER-positive, HER2-negative, and lymph node-positive primary breast cancer. Lymph node positivity was required to be proven histologically prior to the start of treatment with core needle biopsy or fine needle aspiration. All patients were aged between 20 and 50 years. Pre-menopausal status was defined based on the following: (1) last menses within 6 months of randomisation and (2) for patients who have had hysterectomy, E2 ≥ 20 pg/ml and FSH < 30 mIU/ml within 4 weeks of randomisation. ER positivity was defined as Allred score ≥ 3 or modified Allred score ≥ 4. Patients with inflammatory breast cancer, bilateral breast cancer, evidence of distant metastasis, or other malignancy were excluded. Written informed consent was obtained from all participants.

### Randomisation

Patients were enrolled by the study investigators. Patients were randomly assigned (1:1) to receive either NCT or NET for 24 weeks prior to surgery. Block randomisation was performed using the e-CRF system, with patients stratified by the treating centre and clinical stage (stages II and III). Patients who signed an informed consent form entered screening and were assigned a unique patient screening number. Patients who completed the screening process and met all eligibility criteria were randomised through a central randomisation website. No patients were enrolled or began treatment prior to randomisation, and the assignment of a randomisation number was undertaken using the website. Adverse events were recorded at every patient visit and assessed according to the Common Terminology Criteria for Adverse Events Version 3.0.

### Procedures

Patients were randomly assigned (1:1) to receive either adriamycin and cyclophosphamide (60 mg/m^2^ adriamycin plus 600 mg/m^2^ cyclophosphamide intravenously) every 3 weeks for 4 cycles, followed by taxol (75 mg/m^2^ docetaxel intravenously) every 3 weeks for 4 cycles, or goserelin acetate 3.6 mg every 4 weeks with tamoxifen 20 mg daily. Treatment continued for 24 weeks before surgery (Additional file 1).

All patients underwent breast magnetic resonance imaging (MRI) before the start of treatment and after the end of treatment before surgery. Additionally, during treatments (every 3 weeks for the NCT group, and every 4 weeks for the NET group), calliper measurement of the tumour size was performed. We determined the objective tumour response with every measurement method and assessed the response according to the modified RECIST [14]. Surgery was performed between the 24th and 26th week.

All immunohistochemical analyses of ER and Ki-67 expression were performed and reviewed at the central laboratory (Asan Medical Center, Seoul, South Korea). Ki-67 was stained with an antibody for MIB-1 for assessment, using a sample of the core biopsy prior to treatment and a surgery specimen after treatment. The Ki-67 index was calculated as the percentage ratio of Ki-67-positive cells to total cells.

### Outcomes

The primary endpoint was the clinical response rate at 24 weeks, as determined using calliper and MRI measurements. We performed tumour measurements using callipers every 3 weeks for the NEC group and every 4 weeks for the NCT group, and MRI at day 0 and week 24. A clinical response included either a complete response (CR) or a partial response (PR) according to the Response Evaluation Criteria In Solid Tumour (RECIST) version 1.1 criteria. Secondary endpoints were the rate of pCR, the rate of breast conservation surgery, Ki-67 changes, the length of time to maximum response within a treatment period, and QoL, using the European Organisation for Research and Treatment of Cancer Quality of Life (EORTC QOL BR23) questionnaire. Adverse events were recorded at every patient visit and assessed according to the Common Terminology Criteria for Adverse Events Version 3.0.

### Statistical analysis

The sample size was calculated based on the clinical response rate measured by MRI in each group, the NCT and NET groups, under the assumption that the effect of NET would be non-inferior to that of NCT. Sample sizes of 131 in each group achieve 80.055% power to detect a non-inferiority margin difference between the group proportions of − 0.1500 (i.e. the lower limit of the two-sided 95% confidence interval (CI) for the difference in response rates between groups being 15% or less), when the MRI response rate of NCT and NET was estimated to be approximately 75%. A total of 290 patients were required (145 per treatment group, allowing for a drop-out rate of 10% in each group).

Because of enrolment failure, a total of 194 patients were enrolled until September 24, 2014. All data analysis was performed according to a pre-established analysis plan. A two-sided 95% confidence interval for the difference in the incidence of morbidity was calculated to test for non-inferiority. As a non-inferiority design had been adopted, difference of the response rate between group and the 95% CI was calculated, using the index of NCT as the reference. NET would be considered to be non-inferior to NCT when the lower limit of the CI is greater than − 0.15. Because of enrolment failure, a total of 194 patients were enrolled until September 24, 2014. To draw a comparison between the two groups, *t* test for mean differences and chi-squared test for frequencies were used. A 2-sided *p* < 0.05 was considered statistically significant. All analyses were conducted using SAS version 9.4 (SAS Institute; Cary, NC) and R version 3.6.1.

### Role of the funding source

AstraZeneca participated in the funding of the study. The authors had complete access to the study data, and the corresponding author had the final responsibility for publication submission.

## Results

**Fig. 1:**
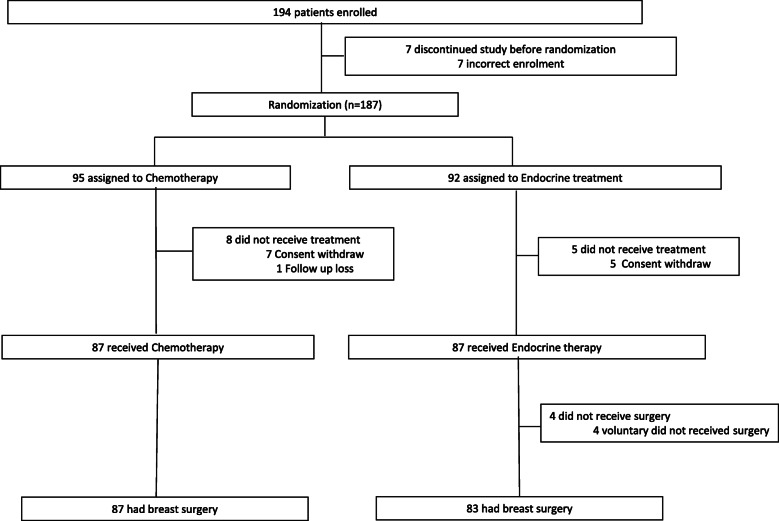
Trial profile

A total of 187 patients from 7 participating centres were included and randomised. Seven patients in the NCT group and 5 patients in the NET group withdraw their consent. One patient in the NCT group was randomised but did not receive treatment. Therefore, a total 174 patients completed the scheduled treatment and were finally analysed (87 patients received NCT and 87 patients received NET; Fig. 1). Four patients in the NET group did not receive the surgery, of whom 3 patients showed PR and 1 patient showed stable disease (SD). The data of these 4 patients were excluded from the post-surgery data (pCR and rate of BCS) analysis (Fig. 1). Baseline characteristics were generally balanced between the treatment groups (Table 1). The median age was 42 years (range, 27–54). All patients were pre-menopausal, and 63% of patients had a normal body mass index. Sixty-nine percent of patients had a clinical T2 breast cancer, and 88.5% had a clinical N1 stage breast cancer. Ninety-four percent of patients had G1/2 breast cancer, and few patients (less than 5%) had poorly differentiated (G3) tumour. The mean Ki-67 expression was not different between the two groups (26.3 for NCT vs. 26.7 for NET, *p* = 0.891). Forty-eight percent of NCT patients and 61% of NET patients were expected to undergo mastectomy prior to the start of treatment (*p* = 0.142).

**Table 1:** Patient demographics and baseline tumour characteristics

	Chemotherapy group (***n*** = 87)	Endocrine therapy group (***n*** = 87)	***p*** value
**Age group at baseline(years)**			
Mean (SD)	42.5 ± 5.6	41.5 ± 5.8	0.255
20–29	2 (2.3%)	2 (2.3%)	
30–39	20 (23.0%)	31 (35.6%)	
40–49	59 (69.0%)	50 (59.8%)	
50–55	6 (5.7%)	4 (2.3%)	
**BMI (kg/m**^**2**^**)**			0.921
< 18.5	5 (5.7%)	4 (4.6%)	
18.5–24.9	54 (62.1%)	56 (64.4%)	
25–29.9	24 (27.6%)	20 (23.0%)	
≥ 30	4 (4.6%)	7 (8.0%)	
**Clinical T stage**			0.746
T1	13 (14.9%)	9 (10.3%)	
T2	58 (66.7%)	62 (71.3%)	
T3	16 (18.4%)	16 (18.4%)	
**Clinical N stage**			0.808
N1	78 (89.7%)	76 (87.4%)	
N2	5 (5.7%)	5 (5.7%)	
N3	4 (4.6%)	6 (6.9%)	
**Grade**			0.616
G1/2	52 (59.8%)	61 (70.1%)	
G3	3 (3.4%)	4 (4.6%)	
NA	32 (36.8%)	22 (25.3%)	
**Ki-67 expression (%)**			0.891
≤ 20%	49 (56.3%)	48 (55.2%)	
> 20%	36 (41.4%)	37 (42.6%)	
Unknown	2 (2.3%)	2 (2.3%)	
**Planned operation**			0.141
Mastectomy	45 (51.7%)	53 (60.6%)	
Breast-conserving surgery	42 (48.3%)	34 (39.1%)	

Data are *n* (%), unless otherwise stated

Tumour size decreased over 24 weeks in both treatment groups (*p* > 0.05). The clinical response rate measured using MRI was 83.7% (72 of 87 patients) with NCT and 52.9% (45 of 87 patients) with NET (estimated difference 30.8%, 95% CI 17.6–44.0, *p* < 0.001). On callipers, the clinical response rate was 83.9% (73 of 87 patients) with NCT and 71.3% (62 of 87 patients) with NET (estimated difference 12.6%, 95% CI 0.4–24.9, *p* = 0.046) (Table 2).

**Table 2:** Summary of clinical response from baseline to week 24

**Clinical response**	**Intention to treat population**	
	**Chemotherapy group** (*n* = 87)	**Endocrine therapy group** (*n* = 87)
**MRI**^+^		
CR	14 (16.3%)	2 (2.3%)
PR	58 (67.4%)	43 (50.6%)
CR + PR	72 (83.7)	45 (52.9%)
Calliper*		
CR	27 (31%)	17 (19.5%)
PR	46 (52.9%)	45 (51.5%)
CR + PR	73 (83.9%)	62 (71.3%)
**Pathologic response**	**Chemotherapy group** (*n* = 87)	**Endocrine therapy group** (*n* = 83)
pCR	3 (3.4%)	1 (1.2%)
pCR (breast)	5 (5.7%)	1 (1.2%)
pCR (axillary lymph node)	12 (13.8%)	4 (4.9%)

Data are *n* (%). *CR* complete response, *PR* partial response

^+^Estimated difference 30.8%, 95% CI 17.6–44.0, *p* < 0.001

*Estimated difference 12.6%, 95% CI 0.4–24.9, *p* = 0.046

The conversion rate of breast-conserving surgery (BCS) with neoadjuvant treatment in patients who planned to have total mastectomy was 13.8% in the NCT group and 11.5% in the NET group, which was not significantly different (*p* = 0.531). Three NCT patients (3.4%) and one NET patient (1.2%) showed pCR (ypT0ypN0M0) (*p* < 0.005). Twelve NCT patients (13.8%) and 4 NET patients (4.9%) had complete lymph node response (*p* < 0.005). However, Ki-67 expression change during the treatment period was not significantly different between the two groups (*p* = 0.114; − 13.56 for NCT and − 7.49 for NET).

Nineteen NCT patients (21.8%) had treatment-related grade 3 or worse adverse events, with no adverse effects in the NET group.

**Fig. 2:**
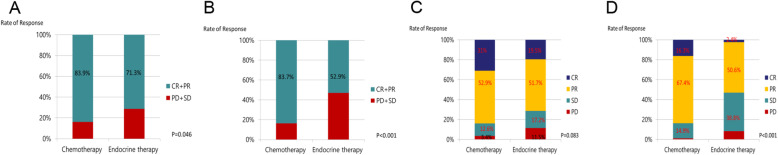
Response rate according to treatments. **a** Clinical response measure by calliper. **b** Clinical response measure by MRI. **c** Detailed clinical response measure by calliper. **d** Detailed clinical response measure by MRI

Exploratory subgroup analysis was performed. There was a difference of tumour response according to Ki-67 expression. For patients with a low Ki-67 expression (≤ 20%), the clinical response rate was 83.7% (41/49) in NCT, 60.4% (29/48) in NET with MRI, and 69.4% (34/49) in NCT, 83.3% (40/48) in NET with calliper. Among the high Ki-67 expression group (> 20%), the response rate was 81.1% (29/36) in NCT, 40.5% (15/37) in NET by MRI, and 77.8% (28 /36) in NCT, 78.4% (29/37) in NET by calliper (Table 3, Fig. 2).

**Table 3:** Summary of clinical response from baseline to week 24

	Treatment group		Low Ki subgroup		High Ki subgroup	
	Chemotherapy arm (*n* = 87)	Endocrine therapy arm (*n* = 87)	Chemotherapy arm (*n* = 49)	Endocrine therapy arm (*n* = 48)	Chemotherapy arm (*n* = 36)	Endocrine therapy arm (*n* = 37)
Best overall tumour response						
MRI						
CR	14 (16.3%)	2 (2.3%)	6 (12.2%)	1 (2.1%)	7 (19.4%)	1 (2.7%)
PR	58 (67.4%)	43 (50.6%)	35 (71.4%)	28 (58.3%)	22 (61.1%)	14 (37.8%)
CR + PR	72 (83.7)	45 (52.9%)	41 (83.7%)	29 (60.4%)	29 (81.1%)	15 (40.5%)
Calliper						
CR	27 (31%)	17 (19.5%)	12 (24.5%)	10 (20.8%)	8 (22.2%)	13 (35.5%)
PR	46 (52.9%)	45 (51.5%)	22 (44.9%)	30 (62.5%)	20 (55.6%)	16 (43.2%)
CR + PR	73 (83.9%)	62 (71.3%)	34 (69.4%)	40 (83.3%)	28 (77.8%)	29 (78.4%)

Data are *n* (%). *CR* complete response, *PR* partial response

Low Ki subgroup: Ki67expression ≤ 20%. High Ki subgroup: Ki67 expression > 20%

## Discussion

During 24 weeks of neoadjuvant treatment, in pre-menopausal, hormone receptor-positive, HER2-negative, and lymph node-positive breast cancer patients, NCT achieved a significantly better clinical response rate than NET. The difference in response was even higher in patients with a highly proliferating tumour (Ki-67 expression > 20). Neoadjuvant therapy was comparatively more effective in patients with low Ki67 (low, 60.4%, vs. high, 40.5%, by MRI) while chemotherapy was equally effective irrespectively of Ki67 (low, 81.1%, vs. high, 83.7%, by MRI).

This study is the first to compare NCT with NET (tamoxifen plus ovarian function suppression) in pre-menopausal breast cancer only. This study is also unique because the patients were all ER-positive/HER2-negative and lymph node-positive.

The primary finding of this study was that the response to NET was inferior to NCT in pre-menopausal ER-positive/HER2-negative subtype breast cancer. The pCR rate was also higher in the NCT group than in the NET group. However, this does not imply that NCT patients will have a better long-term survival outcome than NET patients. First, the changes in Ki-67 expression were not different between the two groups. In addition, NET patients could receive adjuvant chemotherapy in most cases especially when the response was poor (data not shown). Long-term follow-up of the two patient groups is warranted.

Considering pCR and/or clinical response is not a reliable surrogate endpoint for survival, and the most important role of neoadjuvant systemic therapy in ER-positive/HER2-negative breast cancer is expanding the pool of potential BCS candidates by downstaging tumours and permitting BCS in patients who would otherwise require mastectomy [18]. In our study, although NCT led to a better clinical response, the BCS rate was not different between the two groups. Therefore, for the purpose of enabling BCS for mastectomy candidates, NCT might not be a better option than NET.

A potential advantage of neoadjuvant therapy is avoidance of axillary lymph node dissection in patients who have had negative conversion of tumour-positive lymph nodes. NCT downstages axillary nodes in 20 to 40% of patients, and these rates are even higher (> 50%) in HER2-positive patients given anti-HER2 therapy [19–21]. Two recent prospective trials (ACOSOG Z1071 and SENTINA) reported that the false-negative rate was reasonably low when a dual tracer was used and 3 or more sentinel nodes were harvested [22, 23]. Kang et al. showed that in breast cancer patients who had axillary lymph node conversion from clinically positive to negative following NCT, a sentinel lymph node biopsy-guided axillary operation had similar rates of axillary and distant recurrence with axillary lymph node dissection without sentinel node biopsy [24]. Our study showed that for the purpose of avoiding lymph node dissection, NCT can be better than NET because the CR in lymph nodes was significantly higher in the NCT group than in the NET group (13.8% vs. 4.6%). However, further study is needed as to whether complete axillary lymph node dissection has survival benefit and should be a mandatory procedure for patients who have metastasis in a limited number of axillary lymph nodes after NCT or NET.

Most NET studies in breast cancer have focused on post-menopausal women [12, 13, 25, 26]. Few data are available on NET in pre-menopausal women. Some studies have shown that NET could be effective in well-selected pre-menopausal patients [17, 27]. GEICAM has reported randomised phase II results of chemotherapy versus exemestane in pre- and post-menopausal women [28]. Although the sample size was small, the response rate was higher for chemotherapy than for endocrine therapy in pre-menopausal patients, which was consistent with our study. The two therapies were comparable in post-menopausal women and those with a low baseline Ki-67.

The optimal duration of NET is not well defined. Most previous NET studies applied from 3 to 6 months of therapy. In a study by Llombart-Cussac et al. [26], 37% of patients achieved the maximal response beyond 6 months. Carpenter et al. showed that median time to achieve BCS (in those who responded) was 7.5 months [29]. Notably, 62% of panellists at St Gallen 2013 were in favour of continuing NET until a maximal response [30]. In our study, the patients received 6 months of NET. If we had treated to the maximal response for these patients, the response might have been better.

For ER-positive/HER2-negative subtype breast cancer, objective response determination is an issue. pCR, which is the only definite response criteria in neoadjuvant therapy, is highly uncommon; furthermore, it is not a surrogate endpoint for survival in this subtype [31]. We evaluated the clinical response using MRI and callipers. Even though MRI is a useful method for measuring residual tumour size after neoadjuvant treatment, it is not well correlated with pathologic size in ER-positive, HER2-negative tumours [32]. In our study, there was also a large discrepancy between MRI size and pathologic tumour size in the NCT group (data not shown). The difference in response pattern in the tumour between endocrine therapy and chemotherapy makes an objective response determination and comparison difficult and somewhat imbalanced.

There are some limitations in this study. First, the small sample size which was not satisfied with pre-defined number was included. Second, clinical response or pCR is not a predictor of prognosis in ER-positive and Her2-negative subtypes. This data cannot demonstrate the survival benefit of NCT compared with NET. Third, we did not include the arm of aromatase inhibitor as a NET treatment. In a study that compared neoadjuvant GnRH analogue plus tamoxifen and GnRH analogue plus anastrozole in pre-menopausal women, the clinical response was better in the anastrozole group [17]. Because we did not consider an aromatase inhibitor, a question remains concerning the response comparison between chemotherapy versus ovarian function suppression with aromatase inhibitor in pre-menopausal women. Lastly, multigene assays were not evaluated.

Although, in our result for the primary endpoint, NET showed a worse clinical response than NCT, NET cannot be excluded as a clinical option, because (1) the BCS rate was comparable, (2) no difference was observed in Ki-67 change with the therapies, and (3) the adverse event was much smaller in the NET group. This finding indicates that we can recommend NET in clinical practice in ER-positive/HER2-negative breast cancer with low Ki-67 expression, intermediate/low grade tumour, clinically lymph node-negative patients, and for patients with large tumours where BCS is not feasible or equivocal. It is recommended to perform a multigene assay (e.g. Oncotype DX, Mammprint) prior to the start of NET to confirm whether chemotherapy is needed for the individual patient.

## Conclusion

In pre-menopausal breast cancer patients, 24 weeks of NCT showed a better clinical response than NET except for in a subgroup with low Ki-67 or low grade cancer. However, the breast conservation rate and the Ki-67 change were not different. There may be an issue concerning accurate size measurements with MRI in both groups. Clinicians should be aware of the potential benefits and disadvantages of both treatments. Use of NET in pre-menopausal women requires further discussion.

## Supplementary information

**Additional file 1.** Drug administration and Statistics.

## Data Availability

The datasets analysed during the current study are available from the corresponding authors on reasonable request.

## Abbreviations

NET: Neoadjuvant endocrine therapy
NCT: Neoadjuvant chemotherapy
TAILORx: The Trial Assigning Individualised Options for Treatment
RxPonder: Rx for positive node, endocrine responsive breast cancer
MINDACT: Microarray In Node negative Disease may Avoid Chemotherapy
NNBC-3: Node Negative Breast Cancer III Study
ASCO: American Society of Clinical Oncology
SOFT: Suppression of Ovarian Function Trial
TEXT: Tamoxifen and Exemestane Trial
pCR: Pathologic complete response
GnRHa: Gonadotropin-releasing hormone agonist
RECIST: Response Evaluation Criteria In Solid Tumours
IHC: Immunohistochemistry
PEPI: Preoperative endocrine prognostic index
CPS-EG score: Clinic-pathologic stage–oestrogen receptor and tumour grade score
