# Supplementary material for: Efficacy of neoadjuvant endocrine therapy compared with neoadjuvant chemotherapy in pre-menopausal patients with oestrogen receptor-positive and HER2-negative, lymph node-positive breast cancer
Source: Breast Cancer Res. 2020 May 27;22:54. doi: 10.1186/s13058-020-01288-5 (PMC7251809; doi:10.1186/s13058-020-01288-5)
Supplement: Supplementary file 1 — Additional file 1. Drug administration and Statistics. [file 13058_2020_1288_MOESM1_ESM.docx]

Additional file 1. Drug administration and Statistics.

| Dosage and  Administration | **Group A: AC->T**  Adriamycin  Dose: 60 mg/m^2^, D1  Route: Slow intravenous bolus  Schedule: Every 3 weeks  Cyclophosphamide  Dose: 600 mg/m^2^, D1  Route: Slow intravenous bolus  Schedule: Every 3 weeks  Docetaxel  Dose: 75 mg/m^2^, D1  Route: Intravenous, as per local practice  Schedule: Every 3 weeks  **Group B: GnRHa with Tamoxifen**  GnRHa  Dose: 3.6 mg  Route: Subcutaneously, under abdominal skin  Schedule: Every 4 weeks for 6 cycles  A period of 34 days between 2 administrations must not be exceeded.  Tamoxifen  Dose: 20 mg/day  Route: Oral  Schedule: Daily |
| --- | --- |
| Statistical Method | Efficacy  Primary endpoint   - Response rate (according to RECIST v1.1)   Secondary endpoint   - Pathologic complete response - Rate of conservation surgery - Ki-67 changes and its relationship to treatment response - Length of time to maximum response within the treatment period - Tolerability of two treatments - Disease-free survival (DFS) - Overall survival (OS)   PRO (patient reported outcomes using EORTC QLQ-BR23)   - Health state index score - Overall health rating   Safety  Toxicity profile (according to NCI CTCAE v4.0)  Interim Analysis  The interim analysis was performed when 60 participants (30 per group)  had terminated the study treatment. The primary purpose of the  interim analysis was to assess the response rate of the neo-adjuvant  endocrine therapy group. |
